# A cantilevered liquid-nitro­gen-cooled silicon mirror for the Advanced Light Source Upgrade

**DOI:** 10.1107/S1600577520008930

**Published:** 2020-08-11

**Authors:** Grant Cutler, Daniele Cocco, Elaine DiMasi, Simon Morton, Manuel Sanchez del Rio, Howard Padmore

**Affiliations:** a Lawrence Berkeley National Laboratory, 1 Cyclotron Rd, Berkeley, CA 94720, USA

**Keywords:** cantilevered mirrors, wavefront propagation, cryogenics, silicon mirrors, liquid-nitro­gen cooling, high heat-load

## Abstract

This paper presents a novel cantilevered liquid-nitro­gen-cooled silicon mirror design for the first optic in a new soft X-ray beamline that is being developed as part of the Advanced Light Source Upgrade (ALS-U) (Lawrence Berkeley National Laboratory, USA). The beamline is optimized for photon energies between 400 and 1400 eV with full polarization control. Calculations indicate that, without correction, this design will achieve a Strehl ratio greater than 0.85 for the entire energy and polarization ranges of the beamline. With a correction achieved by moving the focus 7.5 mm upstream, the minimum Strehl ratio is 0.99. This design is currently the baseline plan for all new ALS-U insertion device beamlines.

## Abstract 2

A cantilevered liquid-nitrogen-cooled silicon mirror is described that will achieve diffraction-limited performance even under extreme power density. This mirror will serve as a robust first optic for the high-brightness undulator beamlines at the upgraded Advanced Light Source.

## 1. Introduction   

A project to upgrade the Advanced Light Source (ALS) is currently underway. This project (known as the ALS-Upgrade or ALS-U) includes a new soft X-ray ‘FLEXON’ beamline (FLuctuation and EXcitation of Orders in the Nanoscale) optimized for photon energies between 400 and 1400 eV with full polarization control. The current design of this beamline incorporates a 4 m-long apple X-type undulator (Schmidt & Calvi, 2018 ▸), a horizontally deflecting planar first mirror (referred to in this article as M1), a vertically deflecting monochromator, a horizontally deflecting refocusing mirror and an exit slit. This photon delivery system will serve ambitious research programs relying on highly coherent beams, requiring preservation of the wavefront under conditions of exceptionally high power density in the soft X-ray range.

The fundamental challenge in the mechanical design of M1 is to integrate the cooling and mounting system such that the distortion of the mirror – quantified in this paper as the root-mean-square (r.m.s.) height error – is acceptably small for the broad range of heat loads that correspond to the operating range of the undulator. The advantageous material properties of cryogenically cooled silicon and germanium have been well known in the synchrotron light source community since at least 1986 (Rehn, 1986 ▸; Bilderback, 1986 ▸). For high power applications it has been estimated that the slope error for liquid-nitro­gen-cooled silicon is approximately 100 times smaller than for water-cooled silicon (Zhang, 1993 ▸). In the 1990s, this technology was tested in monochromator crystals (Comin, 1995 ▸; Knapp *et al.*, 1995 ▸; Rogers *et al.*, 1995 ▸; Meron *et al.*, 1997 ▸) and it is now used routinely.

A variety of cryogenically cooled silicon monochromator designs have been developed which generally fall into one of two categories: directly or indirectly cooled. Indirectly cooled crystals are clamped between liquid-nitro­gen-cooled copper blocks with a layer of indium foil (Carpentier *et al.*, 2001 ▸; Lee *et al.*, 2001 ▸; Mochizuki *et al.*, 2001 ▸; Tamasaku *et al.*, 2002 ▸; Zhang *et al.*, 2003 ▸; Chumakov *et al.*, 2004 ▸; Zhang *et al.*, 2013 ▸; Huang *et al.*, 2014 ▸). In a directly cooled crystal the liquid nitro­gen is in direct contact with the crystal, which is clamped to a coolant manifold, with the fluid being contained against the silicon by a compressed metal seal (Lee *et al.*, 2000 ▸; Rowen *et al.*, 2001 ▸; Liu *et al.*, 2014 ▸).

Silicon mirrors cooled with liquid nitro­gen are significantly less common, but do exist (Polack *et al.*, 2010 ▸; Brookes *et al.*, 2018 ▸). Considerations in the design of a successful liquid-nitro­gen-cooled silicon mirror system include, but are not limited to, carbon contamination of the optical surface, thermal strain between the mounting system and the mirror, mounting system stiffness, and thermal control, the latter three of which we will now briefly discuss. For a discussion of carbon contamination the reader is referred to Yao-Leclerc *et al.* (2011 ▸), Risterucci *et al.* (2012 ▸), Pellegrin *et al.* (2014 ▸) and Toyoshima *et al.* (2015 ▸). We plan to clean the gold-coated mirror while in operation, using the proven oxygen flow technique (Risterucci *et al.*, 2012 ▸) in which the mirror is exposed to oxygen continuously, and the action of the undulator light is to create reactive radical species that prevent carbon formation. As an alternative, or for implementation of the cryogenically cooled mirror with coatings that are not compatible with oxygen, the mirror chamber can be equipped with *in situ* RF plasma cleaning ports. In this case the mirror temperature would be raised for cleaning and the cleaning gas mixture would depend on the mirror coating (Pellegrin *et al.*, 2014 ▸).

The thermal strain in silicon on cooling from 295 to 125 K is approximately 2.5 × 10^−4^ m m^−1^. To the extent that the mirror mounting structure applies a reaction force to the mirror in response to this contraction, the mirror will deform. Typically, synchrotron beamline optics are kinematically mounted to permit this thermal strain with minimal reaction force. Kinematic mounting can be accomplished with spheres and V-grooves or with flexures; in either case the six degrees of freedom of the optic are exactly constrained and rigid-body motion is prevented. Additionally, differential thermal expansion can be managed by controlling the temperature of the mounting system (Saveri Silva *et al.*, 2017 ▸). However, the stiffness of the mounting system is also important because it partially determines the positional stability of the mirror. The system has to be designed so that vibration does not cause significant intensity noise. The criterion used is that the angular deflection of the mirror is less than 2.5% of the FWHM of the angular source size at the highest energy of the beamline, corresponding to an amplitude noise of 0.1%. For the worst case at ALS-U, this corresponds to an angular amplitude of ∼27 nrad r.m.s. We estimate that, to minimize sensitivity to environmental noise and stay below these vibration limits, the M1 assembly must have a first natural frequency (FNF) above 200 Hz. While this FNF requirement does not necessarily preclude kinematic mounting, designing a sufficiently stiff non-kinematic overconstrained mounting scheme is much more straightforward Another fundamental problem in the design of a kinematically mounted cooled mirror are the forces applied by the coolant lines to the mirror system. These forces vary in magnitude, direction and time with coolant pressure, temperature, flow rate and potentially also mirror alignment, and therefore are not easily characterized. To prevent unwanted motion or distortion of the mirror, the sphere and V-groove based kinematic mounts are often spring-loaded with sufficiently high preload that friction at the sphere to V-groove interface renders the mount non-kinematic. Intentionally non-kinematic designs also exist, one example being the water-cooled cantilever mirror designed for use on an NSLS 1 beamline (Ice & Sparks, 1988 ▸).

Regarding thermal control, ideally the temperature of a cryogenically cooled silicon mirror or crystal is held near 125 K, where the instantaneous coefficient of thermal expansion (CTE) of silicon is approximately zero. To a first-order approximation, the temperature drop from the hottest part of the mirror to the coolant is proportional to the absorbed heat load. In the case of M1 the heat load varies by a factor of approximately five (45 to 220 W), depending on the undulator deflection parameter *K*, and therefore the temperature drop from the peak mirror temperature to the coolant would also vary by a factor of approximately five. One solution is to use electric heaters near the coolant manifold; ideally the extra heat load would be applied on the reflecting surface of the mirror, which could be achieved with the incident X-ray beam itself, by opening an upstream aperture as the *K* of the undulator is reduced. However, as we shall show, in the case of the current M1 neither heaters nor variable apertures are needed, as the optical performance is adequate even at temperatures significantly below 125 K.

**Figure 1:**
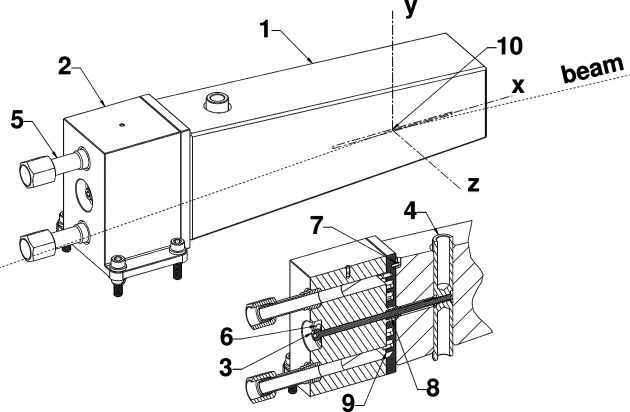
In the ALS-U FLEXON M1, one end of the silicon mirror substrate (labeled 1) is clamped to a cooling manifold (2) with a screw (3) and barrel nut (4). Preload at cryogenic temperatures is maintained by a spring washer (6). Indium foil is compressed between the substrate and manifold. Liquid nitro­gen enters and exits the manifold via the welded-in fittings (5) and flows across an array of pins (9). Movement of the substrate relative to the manifold is prevented by a pair of hollow dowel pins, one in a slot (7) and one in a hole (8). The coordinate system used for finite-element calculations is shown at the center of the beam footprint (10) as a dashed line for the 6σ dimensions of 230 eV photons. The beam grazing angle is 1.25°, which is exaggerated in the drawing for clarity.

In this paper we present the design of a novel liquid-nitro­gen-cooled silicon mirror (Fig. 1 ▸); this end-cooled cantilever design addresses the fundamental challenges of thermal strain, mounting stiffness, unknown coolant line forces and thermal control, as described in the previous paragraph. This paper is divided into two main parts. In the first part we describe our design, outline our analytical approach to thermal tuning and present finite-element calculations of the thermoelastic distortion of the mirror. In the second part we first describe our method for calculating the r.m.s. height error, phase error and Strehl ratio from the finite-element results, and then present wavefront propagation simulations using the deformed mirror shape. Based on these calculations we predict that with a fixed pitch adjustment – but without any higher-order (for example circular) correction – our design will achieve a Strehl ratio greater than 0.85 for the entire operating range of the beamline for two polarization modes. With a correction achieved by adjusting the focal length by 7.5 mm, the minimum Strehl ratio is calculated to be 0.988.

## 2. Description of design   

In the ALS-U FLEXON M1 (Fig. 1 ▸), one end of the silicon mirror substrate is clamped to a manifold made from a nickel–iron Invar alloy. Heat is transferred through the mirror substrate, through a layer of indium foil, across an array of pins machined into the manifold and into the flow of liquid nitro­gen. The mirror is clamped to the manifold by a single screw and barrel nut. The clamping preload is set to achieve the required thermal contact conductance and is maintained at cryogenic temperatures by a spring washer. Translation of the substrate relative to the manifold is prevented by a hollow dowel pin in a hole concentric with the clamping screw, while rotation is prevented by a second pin in a slot.

The idea behind this design is to confine the deformation of the mirror substrate caused by thermal strain of the mirror and its mounting system to an optically insignificant part of the mirror. In other words, the center of the X-ray beam is located sufficiently far from the manifold that strain at the manifold–substrate interface does not significantly affect the shape of the reflecting area. In so doing we are freed from the need to mount the mirror substrate kinematically and can use a comparatively stiff overconstrained mounting system. The stiffness of this mounting system, combined with the tapered shape of the mirror, means that the first natural frequency of the mirror–manifold system is at 402 Hz, a factor of two greater than the design target for ALS-U optics. Thermal control is achieved by tuning the thermal resistance between the mirror substrate and the coolant, and is described in the next section.

### 2.1. Source considerations   

**Figure 2:**
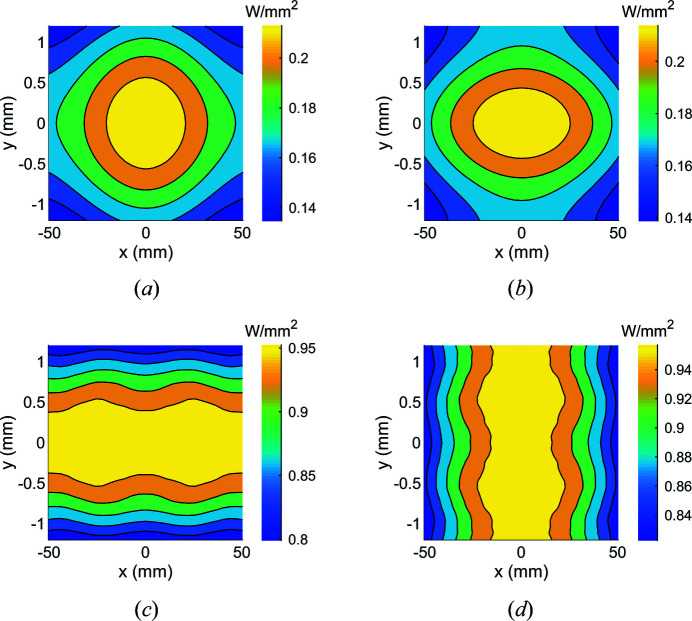
The heat load changes in both magnitude and shape depending on the undulator deflection parameter *K* and the polarization mode. Between *K* = 1 [panels (*a* and (*b*)] and 3 [panels (*c*) and (*d*)] the peak power density ranges from 0.2 to 1 W mm^−2^, while the orientation of the power distribution rotates by 90° between linear horizontal [panels (*a*) and (*c*)] and vertical [panels (*b*) and (*d*)] polarization modes. Note the different axes scales. These heat loads were calculated using the *SPECTRA* code and account for the absorption spectrum of the mirror coating and grazing angle (Tanaka & Kitamura, 2001 ▸).

In the case of M1, both the magnitude and spatial distribution of the absorbed power vary with the undulator deflection parameter *K* and polarization mode (Fig. 2 ▸). To maximize the flux from 400 to 1400 eV, the first harmonic of the undulator radiation would be used from *K* = 2.1 to *K* = 1, with a switch to the third harmonic at ∼874 eV and thereafter using the range *K* = 2.6 to 1.9. For some applications, the beamline will be used down to 230 eV, reached by using the first harmonic to a maximum *K* of 3. The peak power density ranges from approximately 0.2 W mm^−2^ at *K* = 1 to 1 W mm^−2^ at *K* = 3. Over this same *K* range the total absorbed power ranges from 45 to 220 W (assuming a fixed aperture dimension corresponding to ±3 standard deviations of the spatial distribution of 230 eV photons). Additionally, the polarization mode of the undulator can be changed in approximately 3 s. Storage ring, undulator and M1 parameters are given in Table 1 ▸.

### 2.2. Thermal tuning   

**Figure 3:**
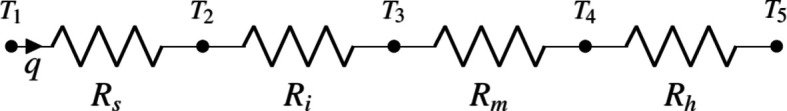
A simple one-dimensional thermal-resistor model of the mirror system. In this model *q* is the heat load and *T*_1_ to *T*_5_ are the temperatures at various locations in the mirror system. The maximum on the mirror surface is *T*_1_, the minimum at the cooled end of the substrate is *T*_2_, the substrate–manifold interface is *T*_3_, the manifold–coolant interface is *T*_4_ and the coolant bulk is *T*_5_. The thermal resistances are: conduction in the mirror substrate *R*_s_, contact resistance at the substrate–manifold interface *R*_i_, conduction in the manifold *R*_m_ and convection to the coolant bulk temperature *R*_*h*_.

We tuned the thermal resistance of our mirror system using a simple one-dimensional thermal-resistor model (Fig. 3 ▸). In this model the thermal resistance of conduction in the mirror substrate *R*_s_ is related to the length *L*_s_, thermal conductivity κ_s_ and cross-sectional area *A*_s_ of the substrate by
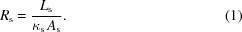
The contact resistance at the substrate–manifold interface is found from
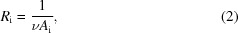
where ν is the thermal contact conductance and *A*_i_ is the interface area. Note that we assume the resistance of conduction across the indium foil to be negligible. The conduction resistance in the manifold is
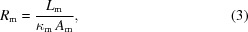
where *L*_m_, κ_m_ and *A*_m_ are, respectively, the length, thermal conductivity and cross-sectional area of the manifold between the interface and the coolant. Finally, the convection resistance is
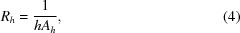
where *h* is the convection film coefficient and *A*_*h*_ is the total manifold–coolant interface area. The temperature drop across the mirror substrate, *T*_1_ − *T*_2_, is
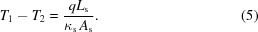
The temperature drop across the substrate–manifold interface is
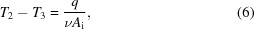
that across the manifold to the coolant interface is
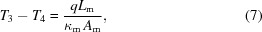
and that from the coolant interface to the coolant bulk is
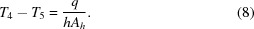
To these ‘design equations’ we also add the temperature rise of the coolant flowing through the manifold at a mass flow rate 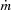,
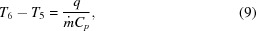
where *C_p_* is the specific heat capacity of the coolant and *T*_6_ is the temperature of the coolant at the manifold outlet. We can choose up to 14 of the 19 variables in these five equations; for the purpose of designing this mirror, it is convenient to choose *T*_1_, *T*_2_, *T*_4_, *T*_5_, *T*_6_, *q*, κ_s_, *L*_s_, ν, *A*_i_, κ_m_, *A*_m_, *A*_*h*_ and *C_p_*, and solve for *A*_s_, *T*_3_, *L*_m_, *h* and 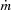. If we assume that the coolant is liquid nitro­gen at *T*_5_ = 77 K, we can choose manifold–coolant interface and manifold outlet temperatures that limit the pressure necessary to prevent vaporization of the coolant, for example *T*_4_ = 80 K and *T*_6_ = 79 K. Because the instantaneous coefficient of thermal expansion of silicon is approximately zero at 125 K, we choose *T*_1_ = 125 K, and because we would like to limit the thermal gradient in the substrate we choose a similar value for the minimum temperature, *T*_2_ = 120 K. For a given undulator *K* and aperture size we know the power *q*. Assuming the substrate is silicon and the manifold is an Invar alloy, we know κ_s_ and κ_m_. Based on published measurements of the thermal contact conductance (Yu *et al.*, 1992 ▸; Asano *et al.*, 1993 ▸; Khounsary *et al.*, 1997 ▸; Marion *et al.*, 2004 ▸) we assume a conservatively low value of ν = 1500 W m^−2^ K^−1^. Once the mass flow rate and convection film coefficient are found, we find the dimensions of the pin array using the model developed by Zukauskas (1972 ▸),
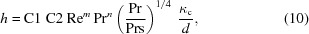
where *C*1, *C*2, *m* and *n* depend on the pin-array geometry, Re is the Reynolds number, Pr is the Prandtl number, Prs is the pin surface Prandtl number, κ_c_ is the thermal conductivity of the coolant and *d* is the pin diameter. Despite its simplicity, this one-dimensional thermal-resistor model agrees with the three-dimensional finite-element model discussed in the next section.

### 2.3. Finite-element calculations of thermoelastic distortion   

**Figure 4:**
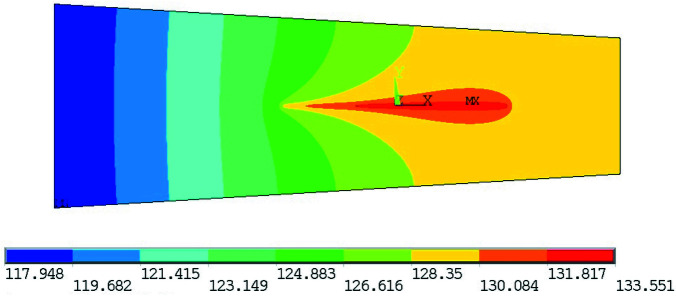
The steady-state temperature of the optically significant portion of the mirror is between 128 and 134 K at *K* = 3 and linear horizontal polarization mode, as computed using the *ANSYS* finite-element code. Image used courtesy of ANSYS, Inc.

**Figure 5:**
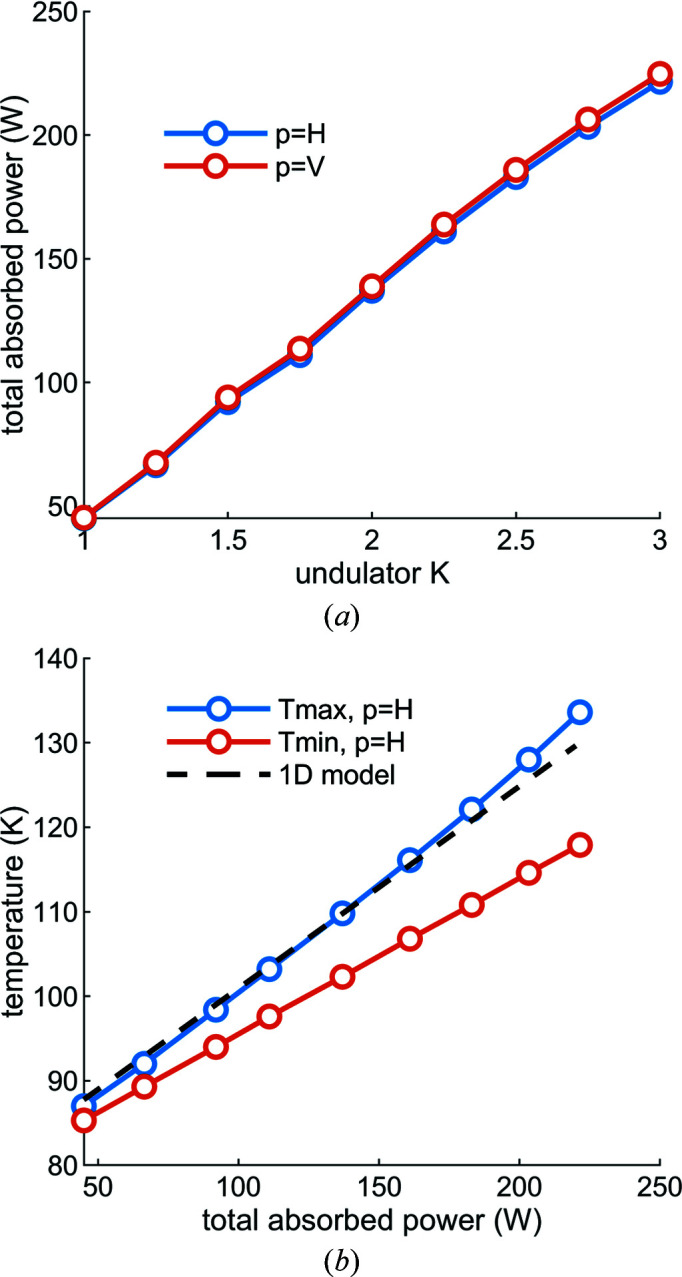
(*a*) The total power absorbed by M1 as a function of undulator deflection parameter *K* is approximately the same for both the linear horizontal (*p* = H) and linear vertical (*p* = V) polarization modes of the undulator. (*b*) The temperature of the mirror as a function of total absorbed power, as calculated with the one-dimensional thermal-resistor model, is in agreement with the three-dimensional finite-element model.

To evaluate the performance of our design we calculated the thermoelastic distortion using the finite-element code *ANSYS* (ANSYS^®^ Mechanical APDL, Release 19.0). In this calculation, the full three-dimensional geometry of the design is modeled, along with temperature-dependent and orthotropic material properties for single-crystal silicon. We modeled the strain at the substrate–manifold interface by fixing the positions of nodes at the interface, as if the substrate were ‘welded’ to a manifold with zero coefficient of thermal expansion. This assumption is conservative because it over-predicts the strain in the substrate. In reality, in cooling from room temperature the manifold contracts, the substrate slides at the interface, and while the indium layer contracts it also permits some internal shear, all of which combine to reduce the overall strain and the resulting thermoelastic distortion of the mirror. To model the strain at the substrate–barrel-nut interface we first computed the local contact pressure with a finely meshed model of the barrel nut and substrate split at the symmetry planes, and then used the resulting pressure distribution as a boundary condition in the substrate model without the meshed barrel-nut geometry. This modeling sequence reduces computational cost and increases accuracy compared with a fine-meshed contact model for the full substrate and nut geometry. We computed the thermoelastic distortion of the mirror for various load steps that simulate the assembly and operation of the mirror. In the first load step we applied a preload tension to the screw, pulling the barrel nut against the substrate. Next we applied gravity, and then cooled the assembly from 295 K to a uniform 77 K. After that we ‘turned on’ the X-ray beam and computed the steady-state temperature distribution and thermoelastic distortion for a range of heat loads corresponding to undulator deflection parameter *K* values between 1 and 3, for both linear horizontal and vertical polarization modes. An example of the temperature distribution is plotted in Fig. 4 ▸. The total power absorbed by M1 as a function of undulator deflection parameter *K* and the temperature of the mirror as a function of total absorbed power are plotted in Fig. 5 ▸.

**Figure 6:**
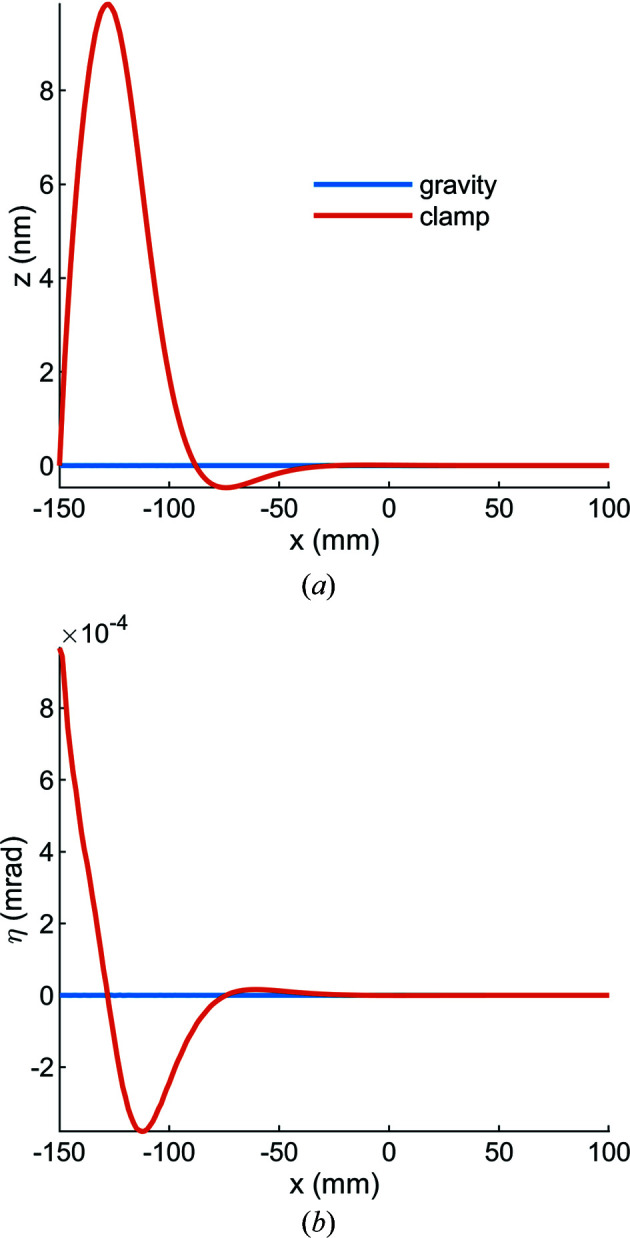
(*a*) The height error and (*b*) the slope error in the tangential plane of the mirror for gravity sag are small compared with that of clamping. In both plots the manifold–substrate interface is at the far left (*x* = −150 mm) and the beam center is at *x* = 0. In panel (*a*), the approximately 10 nm tall bump at *x* = −125 mm is caused by the compression of the barrel nut against the mirror substrate.

**Figure 7:**
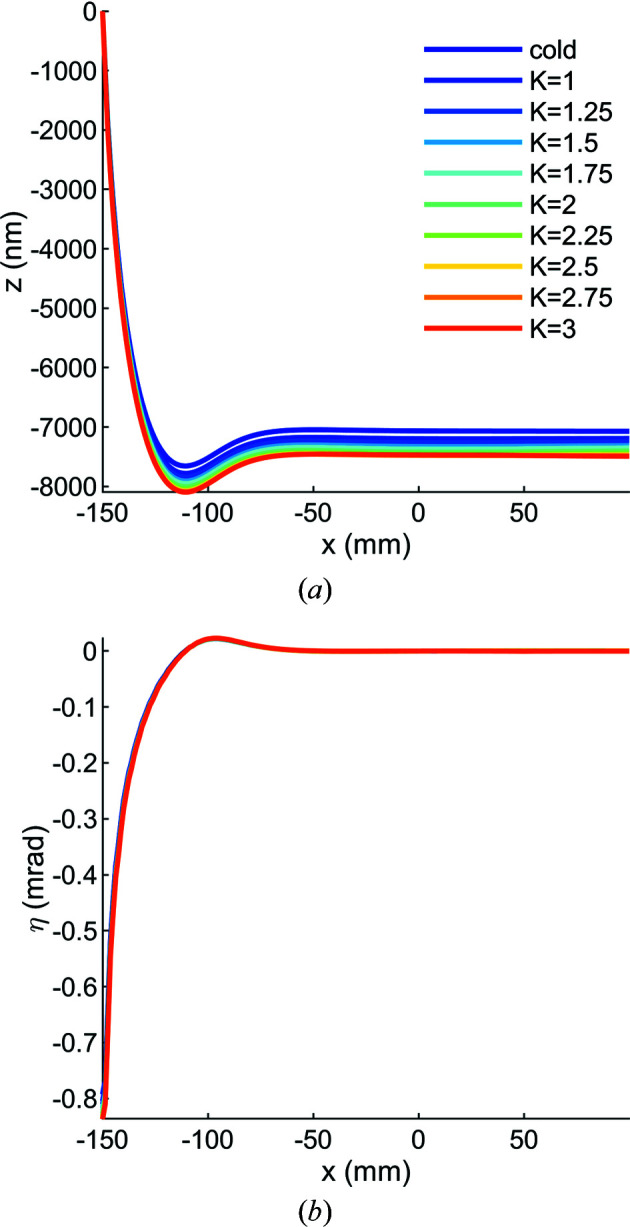
(*a*) As the mirror system is cooled from 295 to 77 K the mirror contracts, resulting in a height error in the tangential plane of approximately −7000 nm. As the undulator *K* value increases from 1 to 3 the contraction continues because the mirror temperature is in the negative CTE regime of silicon. (*b*) To a first-order approximation, the slope error of the mirror in the tangential plane is a constant −0.6 µrad for cool-down and all evaluated undulator *K* values. In both plots the manifold–substrate interface is at the far left (*x* = −150 mm), the beam center is at *x* = 0 and curves are given for the linear horizontal polarization mode of the undulator.

From the results of these simulations we conclude that the thermoelastic distortion is dominated by the cooling step from 295 to 77 K. In other words, not only do clamping and gravity contribute relatively little to the distortion (Fig. 6 ▸), the effect of the X-ray beam power is also small compared with the effect of differential thermal expansion between the manifold and substrate (Figs. 7 ▸ and 8 ▸). This strain causes a ‘cool-down’ pitch in the reflecting portion of the mirror of −0.6 µrad, and this can be corrected either by a rotational stage or by the initial orientation of the mirror during beamline assembly.

## 3. Estimation of height error and Strehl ratio   

We post-processed the finite-element results to calculate the height error, phase error and Strehl ratio. The height error *g* is the root-mean-square (r.m.s.) value inside a window on the mirror’s surface. From the height error, the grazing angle θ and the wavelength λ, we compute the phase error φ from
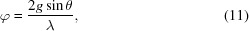
and the Strehl ratio *S* from
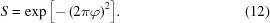



**Figure 9:**
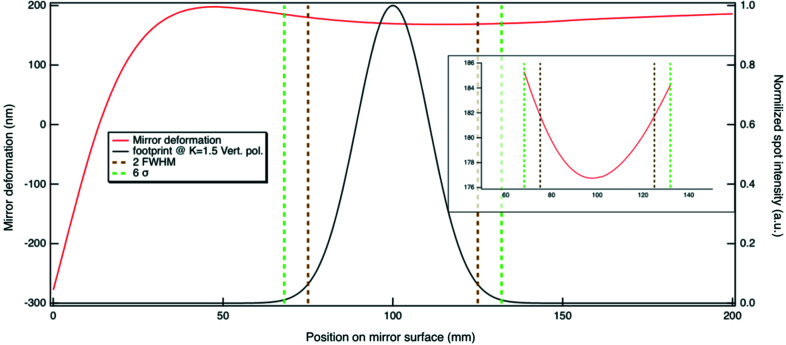
A typical distortion of the mirror over the central 200 mm is plotted, along with the beam footprint on the mirror. The mirror distortion over 6σ with the cool-down pitch removed is shown in the figure inset. The profile of the deformation and the induced wavefront aberration are mostly spherical, and therefore we used the 6σ (dashed green line) window instead of 2 × FWHM (or 4.7σ) (dashed brown lines).

Based on the wavefront propagation simulations described in the next section, we determined that the correct window size for computing the r.m.s. height error is 6σ (six standard deviations) of the (approximately Gaussian) spatial distribution of photons of wavelength λ. This window is larger than the 2 × FWHM (or 4.7σ) window discussed elsewhere (Goldberg & Yashchuk, 2016 ▸; Cocco & Spiga, 2019 ▸) because the height error of this mirror is not random, but instead has a particular profile (Fig. 9 ▸) which mostly affects the spherical and defocus aberrations. The shape of the height error is an important factor in assessing the window size, as also pointed out by Herloski (1985 ▸). In other words, different aberrations should be weighted over different apertures. For example, for coma and astigmatism, 4.8 and 4.7σ are, respectively, the correct window sizes. For spherical aberration the size goes up to 5.6σ. The derivation from Herloski is based on a two-dimensional radially symmetric distribution and provides a guideline for defining the proper window to use for calculating the shape error, phase error and Strehl ratio.

**Figure 10:**
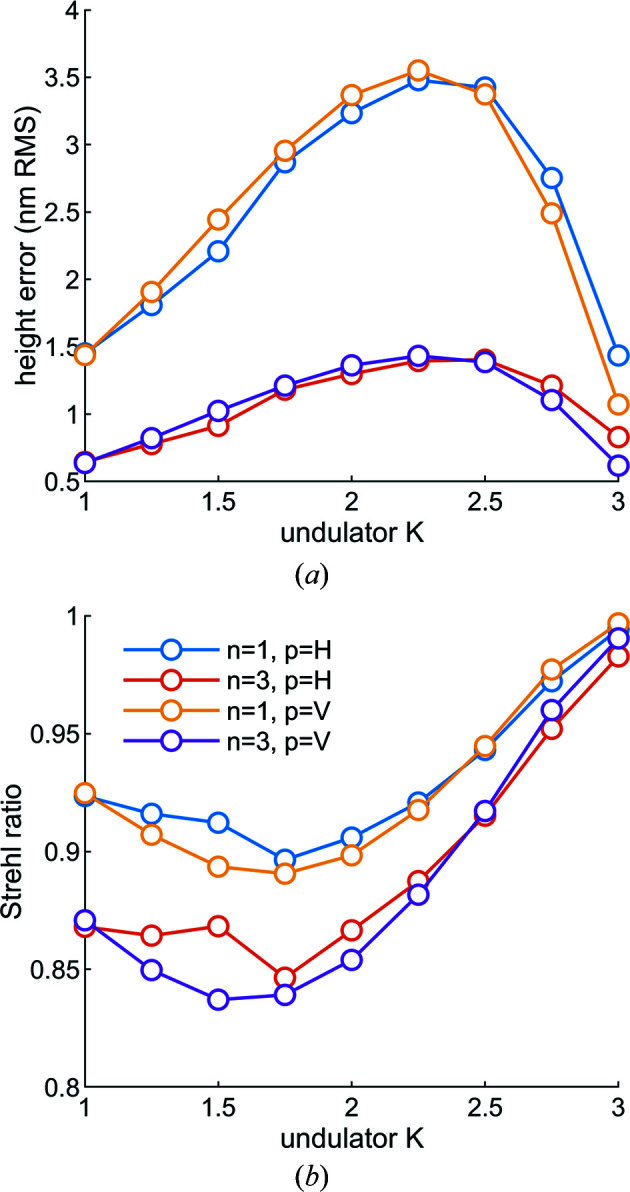
(*a*) The height error and (*b*) the Strehl ratio as a function of undulator deflection parameter *K* for the first (*n* = 1) and third (*n* = 3) harmonics and the linear horizontal (*p* = H) and vertical (*p* = V) polarization modes. Note that while the distortion of the mirror depends only on the undulator *K* and the polarization mode, the r.m.s. window is 6σ of the spatial distribution of the photons of interest, and therefore depends on the harmonic number. The dip in the height error and the corresponding rise in the Strehl ratio at *K* = 1.5 for linear horizontal polarization is due to the nonlinearity of the material properties of silicon combined with the heat-load distribution.

The calculated height error and Strehl ratio are plotted in Fig. 10 ▸. After removing the constant cool-down pitch angle of −0.6 µrad, the height error range is 0.5 to 3.5 nm, and the Strehl ratio range is 0.837 to 0.997. However, to reach the maximum energy of the beamline (1400 eV) the third harmonic is used at *K* = 1.9, where the Strehl ratio is 0.85. The peak Strehl ratio is at *K* = 3, which is to be expected as the mirror system is thermally tuned to be near the zero CTE temperature of silicon at this operating point. At lower *K* values the mirror is colder and the shape error is concave.

### 3.1. Wavefront propagation simulations   

To validate the Strehl ratio calculations and visualize the effect of the thermally induced mirror deformation on the spot size, we simulated a few cases with the wavefront propagation code *WISEr* (Raimondi & Spiga, 2015 ▸) on the open-source platform *OASYS* (Rebuffi & Sanchez del Rio, 2017 ▸; Sanchez del Rio & Rebuffi, 2019 ▸). *WISEr* is a physical optics simulation package which computes the complex electromagnetic field downstream of optical elements. It works across the X-ray spectrum and with grazing angles of incidence, using spatially and temporally fully coherent sources.

We simulated the cases with the lowest Strehl ratios (*K* = 1.5 in first and third harmonics, both polarization modes) and a case that gave a relatively high Strehl ratio (*K* = 2.5, first harmonic and linear horizontal polarization). For the purpose of understanding the effect of mirror deformation on the spot, we performed the simulation in the tangential direction only. Therefore we only considered two mirrors: M1 and the downstream focusing-plane elliptical mirror. M1 is located 13.73 m from the undulator source, while the elliptical mirror is 15.75 m downstream of the flat mirror (source to mirror = 28.752 m) with a focal distance of 4.775 m. Because *WISEr* works with diffraction-limited beams, the dimension at the source was adapted to give the same footprint on M1 as was used in the finite-element model.

**Figure 11:**
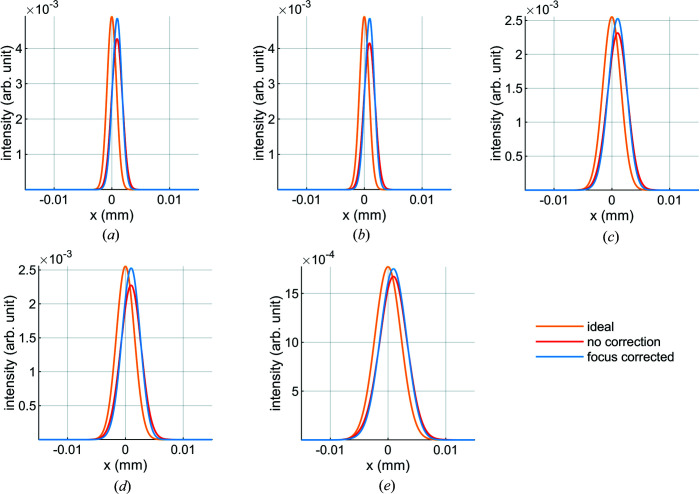
Compared with an ideal mirror (orange curves) the intensity distribution of the thermoelastically deformed M1 (red curves) is shifted horizontally and the peak is lower; the ratio of these peak intensities has been used to calculate the Strehl ratio. By correcting the focus the peak intensity can be increased. The horizontal shift is due to the cool-down pitch of M1 and is independent of *K* value. Distributions are shown for (*a*) *K* = 1.5, third harmonic, linear horizontal polarization, (*b*) *K* = 1.5, third harmonic, linear vertical polarization, (*c*) *K* = 1.5, first harmonic, linear horizontal polarization, (*d*) *K* = 1.5, first harmonic, linear vertical polarization and (*e*) *K* = 2.5, first harmonic, linear horizontal polarization.

**Figure 12:**
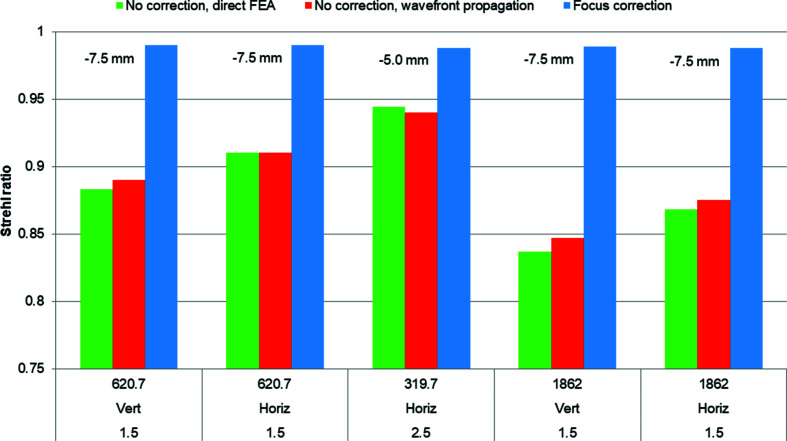
For the thermoelastically distorted M1 without any correction, the Strehl ratio calculated directly from the finite-element analysis (FEA) results (green bars) is in agreement with that calculated using wavefront propagation (red bars) for each of the five studied combinations of photon energy, polarization mode and undulator *K* (for example 620.7 eV, linear vertical polarization, *K* = 1.5). In each case the Strehl ratio can be increased (blue bars) by correcting the shape of the elliptical M3 to move the focus −7.5 mm at *K* = 1.5 and −5 mm at *K* = 2.5, where the negative sign indicates the upstream direction. Note that 1862 eV is outside the optimal range of the FLEXON beamline, but was included in these calculations for comparison of calculation methods.

For the five cases we calculated the spot at the focal location of the elliptical mirror for three conditions: a perfectly flat M1, a thermoelastically distorted M1, and a thermoelastically distorted M1 with the focal distance of the elliptical mirror corrected to minimize the spot profile. Because M1 is concave at low *K* values, the minimum spot dimensions are 5 to 7.5 mm upstream of the ideal focus. We then calculated the Strehl ratio for the thermoelastically distorted M1 with and without correction by comparing the peak intensity with that of the perfectly flat mirror (Fig. 11 ▸). We also compared the FWHM of the intensity distribution – the spot size – for the perfectly flat mirror with that of the distorted M1 with and without focus correction. In all calculated cases, the ratio of the distorted FWHM to the ideal FWHM is the same as the Strehl ratio. The height errors, Strehl ratios, spot sizes and focus corrections are summarized in Figs. 12 ▸ and 13 ▸.

## 4. Summary and conclusions   

In this paper we have presented the novel cantilevered liquid-nitro­gen-cooled silicon mirror design that is being developed as the baseline M1 (or first mirror) for the Advanced Light Source Upgrade (ALS-U). Our calculations indicate that, without correction, this design will achieve a Strehl ratio greater than 0.85 for the entire energy and polarization ranges of the beamline. With a correction achieved by moving the focus 7.5 mm upstream, the minimum Strehl ratio is 0.99. This focal distance change corresponds to about 0.16% of its original value and, if required, can be accomplished with a single-actuator mechanical bender.

Several important conclusions can be made from the results presented in this paper. First, temperatures in the mirror system can be accurately calculated from a one-dimensional thermal-resistor model, which facilitates tuning the system for specified heat loads. Second, in this case it is not strictly necessary to operate the mirror at the so-called ‘sweet-spot’ temperature of silicon (∼125 K), which means that additional heaters or variable apertures are not necessary. Third, the appropriate window size for calculating the r.m.s. height error (and Strehl ratio) for this particular deformation is 6σ of the spatial distribution of photons of the wavelength of interest. Fourth, the Strehl ratio and the increase in spot size (the ratio between the FWHM of the ideal spot and the thermoelastically deformed spot) are in agreement, as expected, because the wavefront aberration is mostly spherical and does not change the Gaussian distribution of the beam. Fifth, although the lowest uncorrected Strehl ratio is 0.85, in reality a Strehl ratio close to or in excess of 0.9 is more than adequate for all the situations we currently envision for the ALS-U FLEXON beamline, especially because the spherical aberration of the mirror does not produce beam striation out of focus. Even for a beamline using imaging techniques, which requires a uniform beam in and out of focus, the presented cryogenically cooled mirror is the ideal solution.

## Figures and Tables

**Figure 8:**
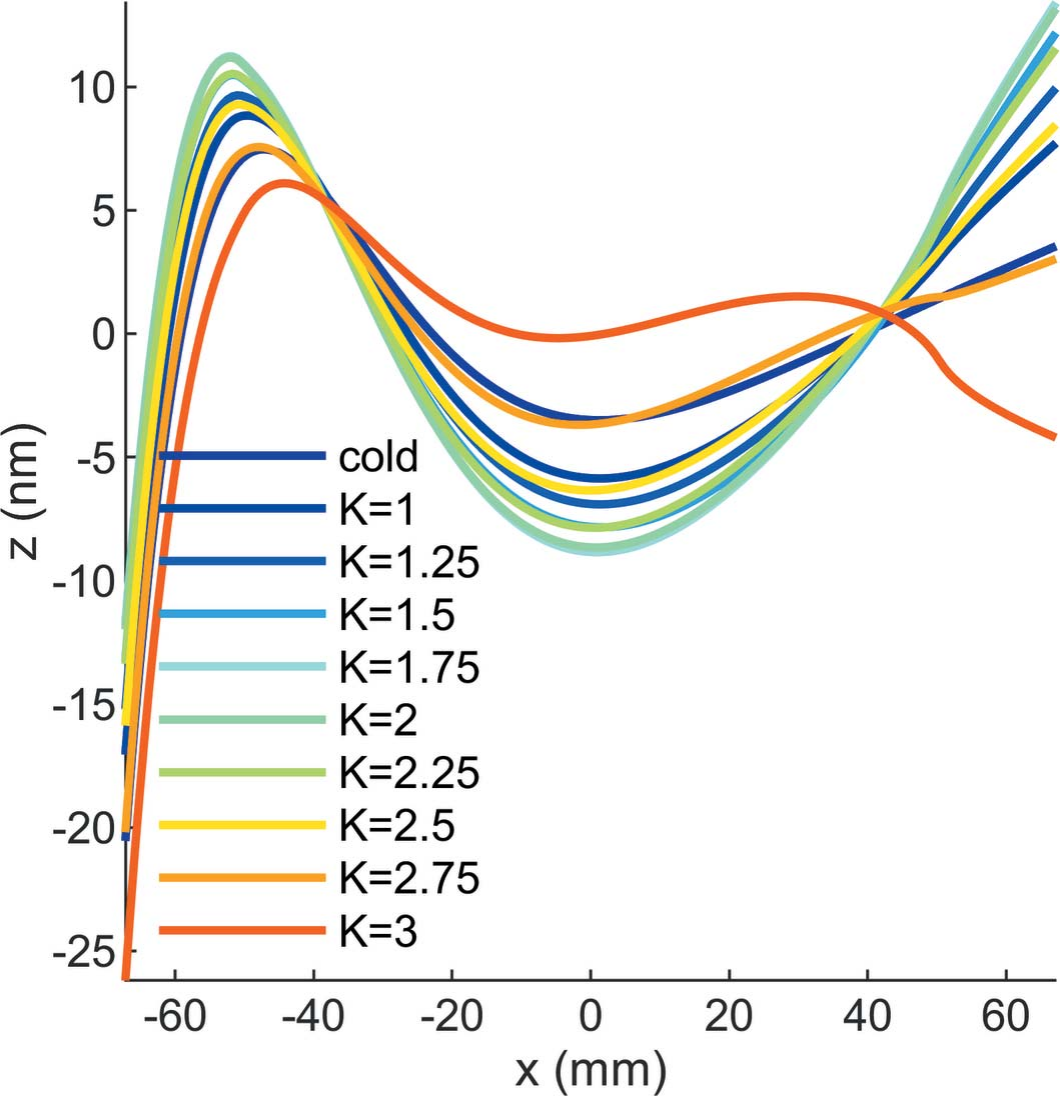
After removing the constant pitch of −0.6 µrad from the tangential-plane height-error curves plotted in Fig. 5 ▸ and zooming in to the central ±70 mm of the mirror, the thermoelastic distortion of the mirror for cool-down from 295 to 77 K (cold) and a range of undulator *K* values can be more easily compared. Because of the thermal tuning of the mirror system, the mirror temperature is entirely in the negative CTE regime for silicon up to *K* = 2.75 and is therefore concave. At *K* = 2.75 the maximum temperature crosses 125 K, which can be seen by the small convex bump at *x* ≃ 40 mm. At *K* = 3 the mirror temperature has increased and the central portion of the mirror begins to flatten. The curves are given for the linear horizontal polarization mode of the undulator.

**Figure 13:**
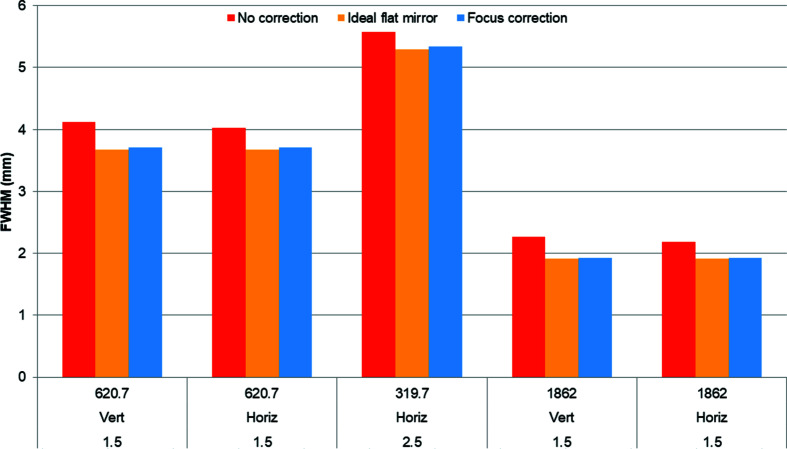
We used a wavefront propagation simulation to compute the FWHM dimension of the spot for the uncorrected thermoelastically distorted M1 (red bars), an ideal flat mirror (orange bars) and after a focus correction (blue bars) for each of the five studied combinations of photon energy, polarization mode and undulator *K* (for example 620.7 eV, linear vertical polarization, *K* = 1.5).

**Table 1:** Parameters for ALS-U storage ring, FLEXON beamline undulator and mirror M1

Parameter	Value	Unit
Storage ring		
Electron energy	2	GeV
Average current	500	mA

Undulator		
Period	28.2	mm
No. of periods	137	

Mirror		
Distance from source	13.73	m
Deflection plane	Horizontal	
Coating	Gold	
Grazing angle	1.25	°
